# Army Orders.—September, 1917. Part III.—Nurses Disabled

**Published:** 1919-10-18

**Authors:** 


### PENSIONS AND GRATUITIES FOR DISABLED NURSES.

### Army Orders.—September, 1917. Part III.—Nurses Disabled.

23. *Disablement Pensions to Nurses.*—(1) A member of Our Queen Alexandra's Imperial Military Nursing Service, of Our Army Nursing Service Reserve, and of Our Territorial Force Nursing Service (hereinafter referred to as a nurse) who retires on account of medical unfitness certified as either attributable to or aggravated by military service in consequence of the present war, and not being due to her serious negligence or misconduct, may be granted the pension shown in the Third Schedule to this Our Warrant, which corresponds to the degree of disablement as certified.

(2) A nurse who is eligible for or in receipt of a pension under the terms of Our Royal Warrant of December 1, 1914, for the Pay, Appointment, Promotion, and Non-effective Pay of Our Army, may be granted (*a*) such pension, together with an addition as shown in the last column of the Third Schedule of this Our Warrant, or (*b*) the pension provided by that Schedule for her rank and degree of disablement, whichever be more favourable.

24. *Temporary Awards to Disabled Nurses.*—(1) If a nurse's disablement is not permanent, the grant of a pension under the foregoing Article shall be temporary, and shall not be made permanent unless the permanency of the disablement is established.

(2) *Permanent Awards to Disabled Nurses.*—When a nurse's pension has been made permanent it shall not be altered on account of any change in her earning capacity, whether resulting from training or other cause, neither shall it be subject to review except when she claims that there has been a substantial increase in the extent of disablement due to the original cause.

25. *Conditions as to Undergoing Treatment.*—Half the pension awarded under the preceding Articles may be subject to the condition that the disabled nurse shall undergo medical treatment in a sanatorium, hospital, convalescent home, or otherwise, for any period during which it is certified that such treatment is necessary in her interests.

26. *Grants to Disabled Nurses Undergoing Treatment.*—A nurse in receipt of a pension under this Our Warrant may be granted in addition to that pension—

(*a*) The difference (if any) between that pension and a pension at the rate for the highest degree of disablement for any period during which she is certified to require special medical treatment in a sanatorium, hospital, convalescent home, or other institution, a deduction of such an amount and under such conditions as Our Minister of Pensions may determine being made from the nurse's pension on account of the cost of her maintenance in the institution;

(*b*) Actual necessary medical and other expenses incidental to treatment in respect of the disability for which she was retired up to such amount and subject to such conditions as Our Minister of Pensions may determine;

(*c*) If disabled in the highest degree an allowance not exceeding £52 a year in any case where the constant attendance of a second person is necessary.

27. *Grants to Disabled Nurses Undergoing Training.*—A nurse in receipt of pension under this Our Warrant may be granted in addition to that pension—

(*a*) The difference, if any, between that pension and pension at the rate for the highest degree of disablement for any period during which she is prevented from earning her living by undergoing training in a technical institution or otherwise which in the opinion of Our Minister of Pensions would benefit her;

(*b*) An allowance to cover fees in respect of training up to such amount and subject to such conditions as the Minister may determine.

28. *Minor Disablement Gratuities to Nurses.*—In any case where the degree of disablement is assessed at less than 20 per cent., or where it is considered by Our Minister of Pensions more in the interests of the nurse, a gratuity or temporary allowance may be granted in place of any pension under this Our Warrant. The grant will be subject to such conditions as Our Minister of Pensions may determine, and its amount will not exceed £300, and will depend on the nature of the disablement and the other circumstances of the case.

29. *Gratuities to Nurses for Disablement not Attributable to or Aggravated by Military Service.*—A nurse who retires on account of medical unfitness certified to be neither attributable to nor aggravated by military service may be granted a gratuity or temporary allowance. The grant will be subject to such conditions as Our Minister of Pensions may determine. In exceptional circumstances it may amount to a sum not exceeding £200, and generally it will depend on the extent to which the nurse is incapacitated and on the other circumstances of the case.

Third Schedule.

| Degree of Disablement (1) | Percentage degree of Disablement (2) | Disablement Pension. If not entitled to Service Pension: Principal Matron or Matron-in-Chief (3) | Matron (4) | Staff Nurse or Sister (5) | Addition to Service Pension if entitled to such (6) |
|---|---|---|---|---|---|
| | Per cent. | £ s. | £ s. | £ | £ s. |
| 1 | 100 | 175 0 | 125 0 | 100 | 75 0 |
| 2 | 80 | 140 0 | 100 0 | 80 | 60 0 |
| 3 | 70 | 122 10 | 87 10 | 70 | 52 10 |
| 4 | 60 | 105 0 | 75 0 | 60 | 45 0 |
| 5 | 50 | 87 10 | 62 10 | 50 | 37 10 |
| 6 | 40 | 70 0 | 50 0 | 40 | 30 0 |
| 7 | 30 | 52 10 | 37 10 | 30 | 22 10 |
| 8 | 20 | 35 0 | 25 0 | 20 | 15 0 |

